# Analysis of CT signs, radiomic features and clinical characteristics for delta variant COVID-19 patients with different vaccination status

**DOI:** 10.1186/s12880-022-00937-9

**Published:** 2022-11-29

**Authors:** Huanhuan Wei, Zehua Shao, Jianqing Tai, Fangfang Fu, Chuanjian Lv, Zhiping Guo, Yaping Wu, Lijuan Chen, Yan Bai, Qingxia Wu, Xuan Yu, Xinling Mu, Fengmin Shao, Meiyun Wang

**Affiliations:** 1 Academy of Medical Sciences, The People’s Hospital of Zhengzhou University, 7 Weiwu Road, Zhengzhou, 450003 Henan China; 2 Heart Center of Zhengzhou University People’s Hospital, Henan Provincial People’s Hospital, Zhengzhou, 450003 Henan People’s Republic of China; 3 The First People’s Hospital of Zhengzhou, 56 East Street, Zhengzhou, 450004 Henan China; 4 Department of Medical Imaging, Henan Provincial People’s Hospital & the People’s Hospital of Zhengzhou University, 7 Weiwu Road, Zhengzhou, 450003 Henan China; 5 Beijing United Imaging Research Institute of Intelligent Imaging, Beijing, 100089 China

**Keywords:** COVID-19 vaccine, Radiomics, Computed tomography

## Abstract

**Objective:**

To explore the characteristics of peripheral blood, high resolution computed tomography (HRCT) imaging and the radiomics signature (RadScore) in patients infected with delta variant virus under different coronavirus disease (COVID-19) vaccination status.

**Methods:**

123 patients with delta variant virus infection collected from November 1, 2021 to March 1, 2022 were analyzed retrospectively. According to COVID-19 vaccination Status, they were divided into three groups: Unvaccinated group, partially vaccinated group and full vaccination group. The peripheral blood, chest HRCT manifestations and RadScore of each group were analyzed and compared.

**Results:**

The mean lymphocyte count 1.22 ± 0.49 × 10^^9^/L, CT score 7.29 ± 3.48, RadScore 0.75 ± 0.63 in the unvaccinated group; The mean lymphocyte count 1.55 ± 0.70 × 10^^9^/L, CT score 5.27 ± 2.72, RadScore 1.03 ± 0.46 in the partially vaccinated group; The mean lymphocyte count 1.87 ± 0.70 × 10^9/L, CT score 3.59 ± 3.14, RadScore 1.23 ± 0.29 in the fully vaccinated group. There were significant differences in lymphocyte count, CT score and RadScore among the three groups (all *p* < 0.05); Compared with the other two groups, the lung lesions in the unvaccinated group were more involved in multiple lobes, of which 26 cases involved the whole lung.

**Conclusions:**

Through the analysis of clinical features, pulmonary imaging features and radiomics, we confirmed the positive effect of COVID-19 vaccine on pulmonary inflammatory symptoms and lymphocyte count (immune system) during delta mutant infection.

## Introduction

Since the outbreak of unknown pneumonia in Wuhan at the end of 2019, the pandemic caused by COVID-19 and severe acute respiratory syndrome coronavirus-2 (SARS-CoV-2)-related disease has brought great challenges to human beings all over the world, and it has caused tens of thousands of deaths around the world and economic problems in many countries [1, 2]. delta mutant strain appeared during the second wave of infection in India in October 2020 [3]. Different from novel coronavirus's original strain, the transmission capacity of delta strain was about 40% higher than that of the original strain, and it had the characteristics of short incubation period and interval, high virus load and easy to develop into critical illness [4–7]. Therefore, it spreads rapidly internationally and is still evolving.

The world health organization (WHO) has announced that COVID-19 has become a public health event of international concern. In the light of the actual situation of the local epidemic, local governments have introduced and urged the implementation of a series of major measures for the prevention and control of the epidemic, among which vaccination is considered to be one of the most effective ways to prevent infectious diseases, which can reduce the hospitalization rate of patients, the severity of the disease and even the fatality rate. At present, there are 3 kinds of vaccines that are commonly vaccinated, namely (1) Inactivated vaccines (2-injection immunization), the vaccines prepared by a series of purification techniques after killing the cultured and amplified live virus by physical and chemical methods. The composition is similar to the natural virus structure, the immune response is relatively strong, and it has good safety and stability [8]; (2) Recombinant protein vaccine (3-injection immunization) is the most effective antigen component through genetic engineering. Vaccines obtained through protein expression and purification process have a relatively low adverse reaction rate [9]. (3) Adenovirus vector vaccine (1-shot immunization), which uses type 5 adenovirus as a carrier, introduces the new coronavirus antigen gene, and makes a live vector vaccine through a bioreactor. The vaccine can well induce antibody production and enhance cellular immunity [10].

To date, the gold standard for the diagnosis of delta strain pneumonia is still real-time reverse transcription polymerase chain reaction (RT-PCR). Additionally, chest CT also plays an important role in diagnosing suspected delta coronavirus disease cases with the advantage of higher prior probability [11]. In order to improve the efficiency of CT in detecting lesions under the premise of low misdiagnosis rate, we introduce radiomics, which can overcome visual instability and omission of a large amount of image information [12, 13]. Recently, some studies have proved the value of radiology based on CT in the diagnosis, differential diagnosis and follow-up of coronavirus pneumonia [14, 15]. However, to the best of our knowledge, there are few reports on the use of chest CT radiomics features to study the value of COVID-19 vaccine. Thus, the peripheral blood laboratory test indicators, chest CT scores and radiomics features of the unvaccinated population, the population partially vaccinated, and the population fully vaccinated against the COVID-19 vaccine were compared and analyzed in order to investigate the protective effect of the COVID-19 vaccine on delta variant strain infection and provide more data and decision support for accurate strategies for epidemic prevention and control.

## Methods

This retrospective study was approved by the Ethics Committee of Henan Provincial People’s Hospital, and the requirement for informed consent was waived.

### Study population

A total of 150 consecutive patients with delta mutant coronavirus disease between November 1, 2021 and March 1, 2022 were enrolled. The diagnosis of delta coronavirus disease was based on the Guidelines for the Diagnosis and Treatment of Novel Coronavirus Pneumonia (Trial Eighth Revision) proposed by the National Health Committee of the People's Republic of China in 2022 [16]. Inclusion criteria (1) novel coronavirus nucleic acid detection was positive; (2) Delta variant strains were identified by high-throughput whole genome sequencing; (3) Novel coronavirus had a clear history of vaccination, and the time of CT examination and the last vaccination was no more than 6 months. Exclusion criteria: (1) poor CT image quality; (2) patients with severe pulmonary comorbidities, such asobstructive pulmonary emphysema; (3) patients with unknown vaccination history or patients aged < 18 years; Finally, a total of 123 patients met the criteria were included, and all included patients were divided into unvaccinated group, incompletely vaccinated group and fully vaccinated group according to COVID-19 vaccination Status, vaccine types including inactivated vaccines, recombinant protein vaccine and adenovirus vector vaccine. The peripheral blood laboratory tests of the selected cases were completed on the day of admission, and the first chest CT was completed in no more than a week.

### CT images acquisition

All patients were scanned with non-contrast enhanced HRCT (uCT760, Shanghai, China). The specific CT scanning parameters are as follows: Scan slice thickness 5 mm, pitch 0.9, 120 kV, and 180 mA. All scans were performed with the patient supine and, where possible, taking into account the patient's breath holding and sufficient inhale to obtain the best quality image from the tip of the lung to the bottom of the lung. In order to optimize the signal-to-noise ratio, the standard algorithm post-processing is used for image reconstruction after the scanning is completed.

### Laboratory biochemistry tests

The patients infected with delta variant strains were given isolation treatment and symptom treatment, including maintenance of internal environment stability, anti-infective treatment and nutritional support. According to the changes of oxygen saturation, timely effective oxygen therapy measures, including nasal catheter, mask oxygen, if necessary, nasal high flow oxygen therapy, non-invasive or invasive mechanical ventilation and so on. The following data are derived from the electronic medical record system (Table 1). This includes key clinical features (sex, age, vaccination) and peripheral blood laboratory tests, which are reviewed by a clinician.

**Table 1:** Demographic information and results of laboratory tests in patients in patients infected with delta variant virus

	Unvaccinated group(*n* = 41)	Partially vaccinated group(*n* = 41)	Full vaccination group(*n* = 41)	F/χ2 value	*P* value
Sex (male, %)	19(46.34%)	22(53.66%)	20(48.78%)	0.20	0.91
Age (years, ${\bar{\text{x}}} \pm {\text{s}}$)	61 ± 22.96	44.93 ± 16.58^*^	54.15 ± 15.03^*^	8.43	< 0.001
White Blood Cell Count (× 10^9^/L)	5.96 ± 2.54	5.70 ± 1.90	5.85 ± 1.73	0.157	0.855
Lymphocyte Count (× 10^9^/L)	1.22 ± 0.49	1.55 ± 0.70^*^	1.87 ± 0.70^*†^	10.78	< 0.001
CT score	7.29 ± 3.48	5.27 ± 2.72^*^	3.59 ± 3.14^*†^	14.41	< 0.001
RadScore (Median ± quartile)	0.75 ± 0.63	1.03 ± 0.46^*^	1.23 ± 0.29^*†^	21.72	< 0.001

*Compared with the unvaccinated group, *P* < 0.05; ^†^ Compared with partially vaccinated group, *P* < 0.05

### Image analysis and CT scores

Two radiologists (with 10- and 9-years’ experience in chest radiology, respectively) independently reviewed all CT images in the medical imaging system, and if there were any differences in lesion assessment, consulted a third senior radiologist (with 25 years of experience in chest radiology) and finally reached an agreement. All evaluators were unaware of the patient's clinical data and the vaccination status of the COVID-19 vaccine. The extent of involvement of each lobe (two lobes in the left lung and three lobes in the right lung) was assessed and classified according to the following factors: no involvement corresponding to a score of 0 (0%), minimal involvement considered minimal (1–25%) and assessed as 1, mild involvement is rated as 2 (26–50%), moderate involvement was scored as 3 (51–75%) and severe involvement was scored as 4 (76–100%). The total CT image score is a total score for the five lobes, ranging from 0 (no involvement) to 20 (maximum involvement), providing the overall severity of lung involvement [17].

We used a commercial software, uAI Research Portal V1.1 (United Imaging Intelligence, Co, Ltd.) to automatically segment the infected pulmonary regions. (Previous scholars have performed regional segmentation and quantitative analysis of lung infection through this platform, and have achieved good results [18]).We defined these regions as 3-dimensional regions-of-interest (3D-ROI). Then we used 3D-ROI to extract radiomics features in the same platform, which embedded Python (version 3.7.3) and PyRadiomics (version 2.2.0) [19]. In order to reduce the influence of different CT scanning equipment, the image was resampled by setting 3 mm × 3 mm × 3 mm voxel spacing and was normalized by z-score normalization, and two groups of radiological features were obtained: (1) First-order statistical characteristics, reflecting the imaging intensity changes of lung volumes by describing the distribution pattern of image gray values, mainly including mean, median, maximum, minimum, entropy and bias; (2) Texture feature, the change of image gray level related to spatial statistics, reflects the spatial relationship between each voxel and its adjacent voxels from the microscopic level. Common texture features include gray co-occurrence matrix, autoregressive texture model, Tamura texture feature, wavelet transform and so on.

After feature extraction, we first used z-score to normalize the radiomics features. Then we remove the features with variance less than 0.05, and then we used analysis of variance (ANOVA) F-value (*P* < 0.05) to filter out redundant features. Finally, we used the least absolute shrinkage and selection operator (LASSO) regression model to remove the features with high collinearity, and select the most discriminative radiomics features [20]. The optimized hyperparameter α of LASSO is obtained by using fivefold cross-validation, and the features with non-zero coefficients are selected and the RadScore is calculated by multiplying the respective features by the corresponding coefficients.

### Statistical analysis

All data were analyzed using SPSSv.26.0 (SPSS Company, Chicago, Illinois, USA). Continuous quantitative data in accordance with normal distribution are represented by means and standard deviation ($\bar{\text{x}} \pm {\text{SD}}$). Single factor analysis of variance is used for comparison between groups when variance is homogeneous, and the least significant difference test is used for pairwise comparison. When the variance was uneven, Welch analysis of variance was used for comparison among groups, and Tamhane'sT2 test was used for pairwise comparison. The classified variables that do not conform to the normal distribution are represented by the corresponding counting mode and percentage. The Krushal-wallis H test was used to compare the differences among the groups. The parameters with statistical significance were analyzed by the receiver operating characteristic (ROC) curve and the area under the curve (AUC) was calculated. For the fusion model, we used R software (version 3.4.1; Fig. http://www.Rproject.org), performed statistical analysis and plotted the ROC curve and nomogram. since our study is a multi-categorial problem, we performed one-versus-rest ROC analysis of the fusion model across the unvaccinated, partially vaccinated and fully vaccinated groups. Intra-group correlation coefficient (ICC) was used to evaluate the reliability of CT radiological feature score among different observers. When the ICC value is > 0.75, it is considered that the interobserver agreement for the overall chest CT score is good.

## Results

### Clinical baseline characteristics and selected laboratory tests

A total of 123 patients (age: 53.59 ± 16.61) were included, 57 (46.34%) were male and 66 (53.66%) were female. They were divided into three groups according to the status of COVID-19 vaccine: unvaccinated group (41, age 61 ± 22.96), incomplete vaccinated group (41, age 44.93 ± 16.58) and complete vaccinated group (41, age 54.15 ± 15.03). The detailed clinical data are shown in Table 1. Overall difference in age across three groups, the age of the unvaccinated group was older (*p* < 0.05). There were no overall significant differences in gender across the three groups (*p* > 0.05).

### CT score and the findings of CT images

**Fig. 1:**
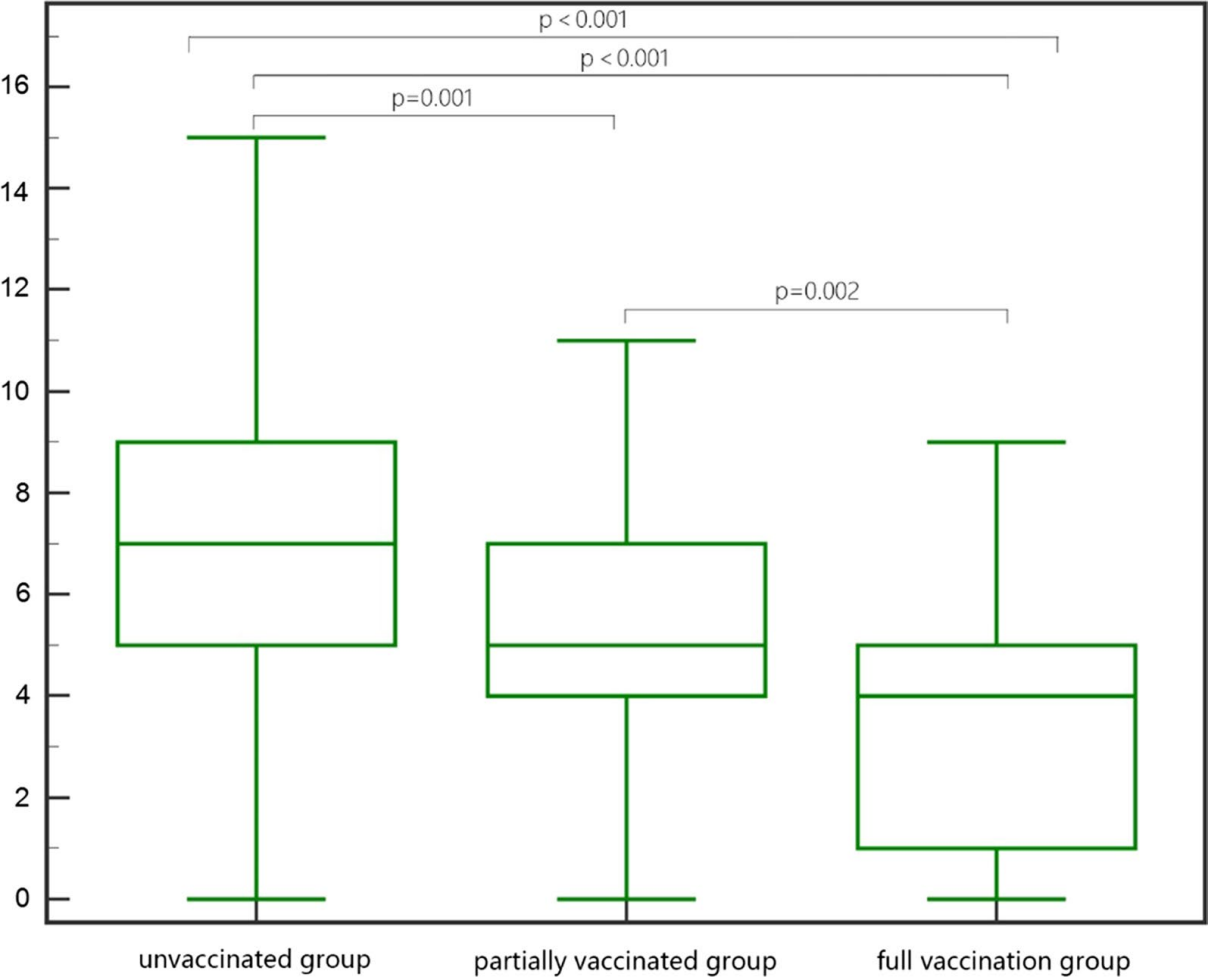
CT score box diagram of unvaccinated group, partially vaccinated group and full vaccination group

The ICC of the CT score between the two physicians was 0.936 (95% confidence interval 0.910 ~ 0.954); In the unvaccinated group, the partially group, and the fully vaccinated group, there were 1, 4, and 7 patients with delta variant infection without abnormal chest CT scans, respectively. The total score of lung involvement in the three groups ranged from 0 to 15. The mean value of unvaccinated group was 7.29 ± 3.48, which was significantly higher than that of partial vaccination group (5.27 ± 2.72) and complete vaccination group (3.59 ± 3.14), and there were significant differences between non-vaccination group and partial vaccination group, between non-vaccination group and complete vaccination group, and between partial vaccination group and complete vaccination group (all *P* < 0.05) (Table 2) (Fig. 1).

**Table 2:** CT scores and chest CT imaging performance of patients under different COVID-19 vaccination status

	Unvaccinated group		Partially vaccinated group		Full vaccination group	
CT score	*n*	(%)	*n*	(%)	*n*	(%)
0 (normal)	1	2.44	4	9.76	7	17.07
1–2 (minimal)	8	19.51	10	24.39	15	36.59
3–5 (mild)	10	24.39	18	43.90	12	29.27
6–10 (moderate)	19	46.34	7	17.07	6	14.63
11–20 (severe)	3	7.32	2	4.88	1	2.44
*Affected lung*						
Right upper lobe	33	89.19	24	64.86	18	54.55
Right middle lung	30	81.08	26	70.27	12	36.36
Right lower lung	36	97.30	27	72.97	25	75.76
Left Upper lung	29	78.38	24	64.86	16	48.48
Left lower lung	34	91.89	30	81.08	19	57.58
All lobes affected	26	63.41	15	36.59	9	21.95
*Distribution*						
Peripheral	20	54.05	23	62.16	19	57.58
Central	0	0	0	0	2	6.06
Peripheral and central	17	45.95	14	37.84	12	36.36
*Opacity*						
Ground glass	16	43.24	19	51.35	17	51.52
Consolidation	3	8.11	0	0	3	9.09
Ground glass shadows with consolidation	18	48.65	18	48.65	13	39.39

**Fig. 2:**
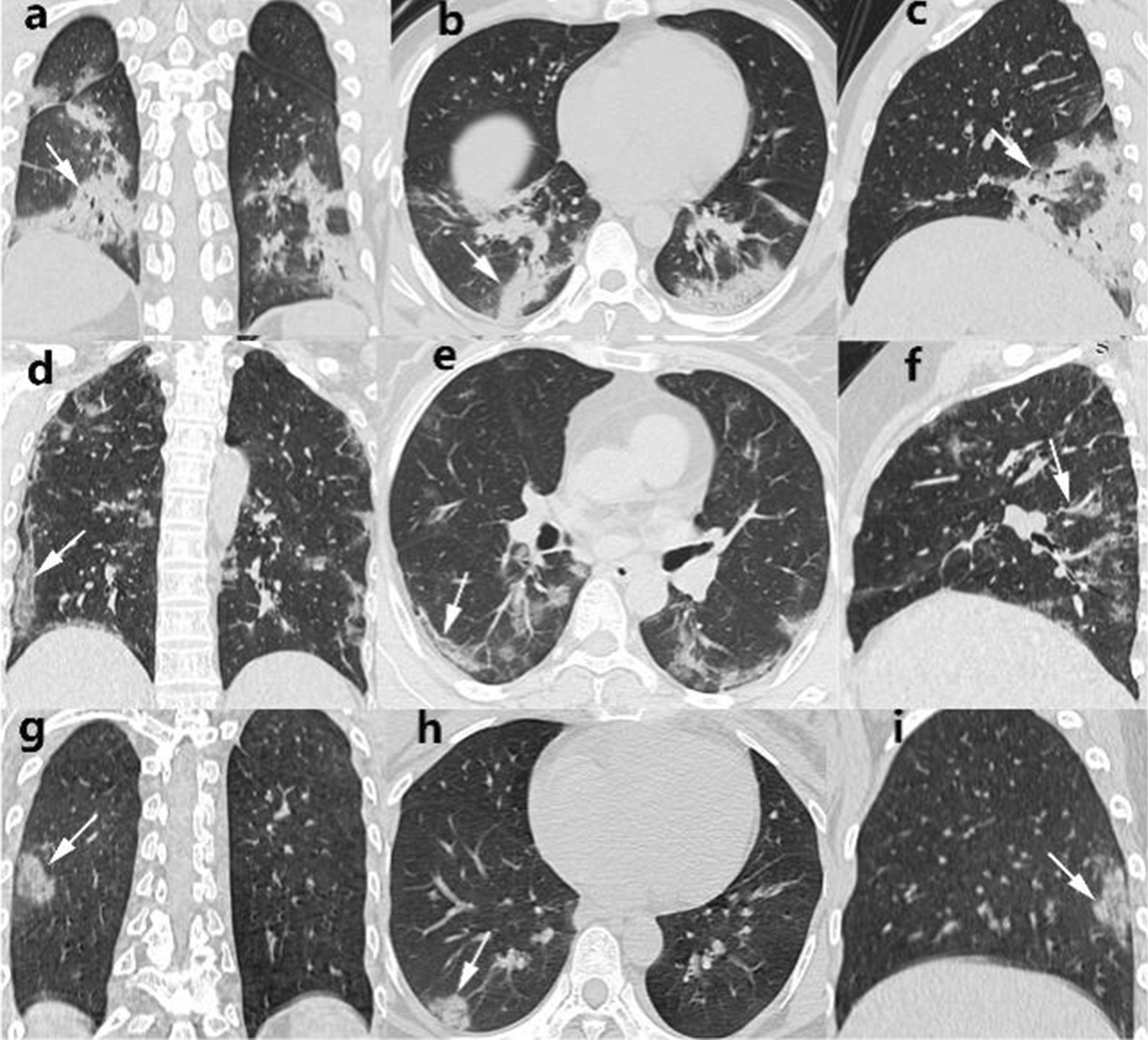
Thin-section CT images of patients infected with Delta variant strains who were unvaccinated with the COVID-19 vaccine, partially vaccinated with the COVID-19 vaccine, and full vaccination with the COVID-19 vaccine. **a-c** Images of A 52-year-old woman, who was not vaccinated with the new coronary vaccine Delta variant strain (CT score of 12), showed diffuse large areas of ground glass shadows in both lungs on coronal, axial, and sagittal CT images, with lesions distributed mainly in both lower lungs, accompanied by consolidation (white arrows). **d-f** Images of a 43-year-old man infected with Delta variant virus who was partially vaccinated (CT score of 6). Coronal, axial, and sagittal CT images all presented scattered ground glass shadows in both lower lobes and subpleura, some with consolidation (white arrows). **g-i** Images of a 49-year-old woman infected with Delta variant virus who was fully vaccinated (CT score of 6). Coronal, axial, and sagittal CT images all presented scattered ground glass shadows in both lower lobes and subpleura, some with consolidation (white arrows). Coronal, axial and sagittal CT images showed subpleural ground glass shadows in the posterior basal segment of the right inferior lobe, with local consolidation (white arrow)

A total of 111cases (90.24%) showed positive results by CT scan. In the non-vaccinated group, one case showed negative CT. Among the 40 patients with positive CT, the lesions mostly involved all lobes of both lungs, including 33 cases (89.19%) in the upper lobe of the right lung, 30 cases (81.08%) in the middle lobe of the right lung, 36 cases (97.30%) in the lower lobe of the right lung, 29 cases (78.38%) in the upper lobe of the left lung, 34 cases (91.89%) in the lower lobe of the left lung and 26 cases (63.41%) in the whole lobe (Fig. 2).

In the three groups, the main pulmonary lesions were ground glass shadows with consolidation or simple ground glass shadows. Most of the lesions were located in the peripheral or subpleural type, followed by the mixed peripheral and central type (Table 2).

### RadScore

**Fig. 3:**
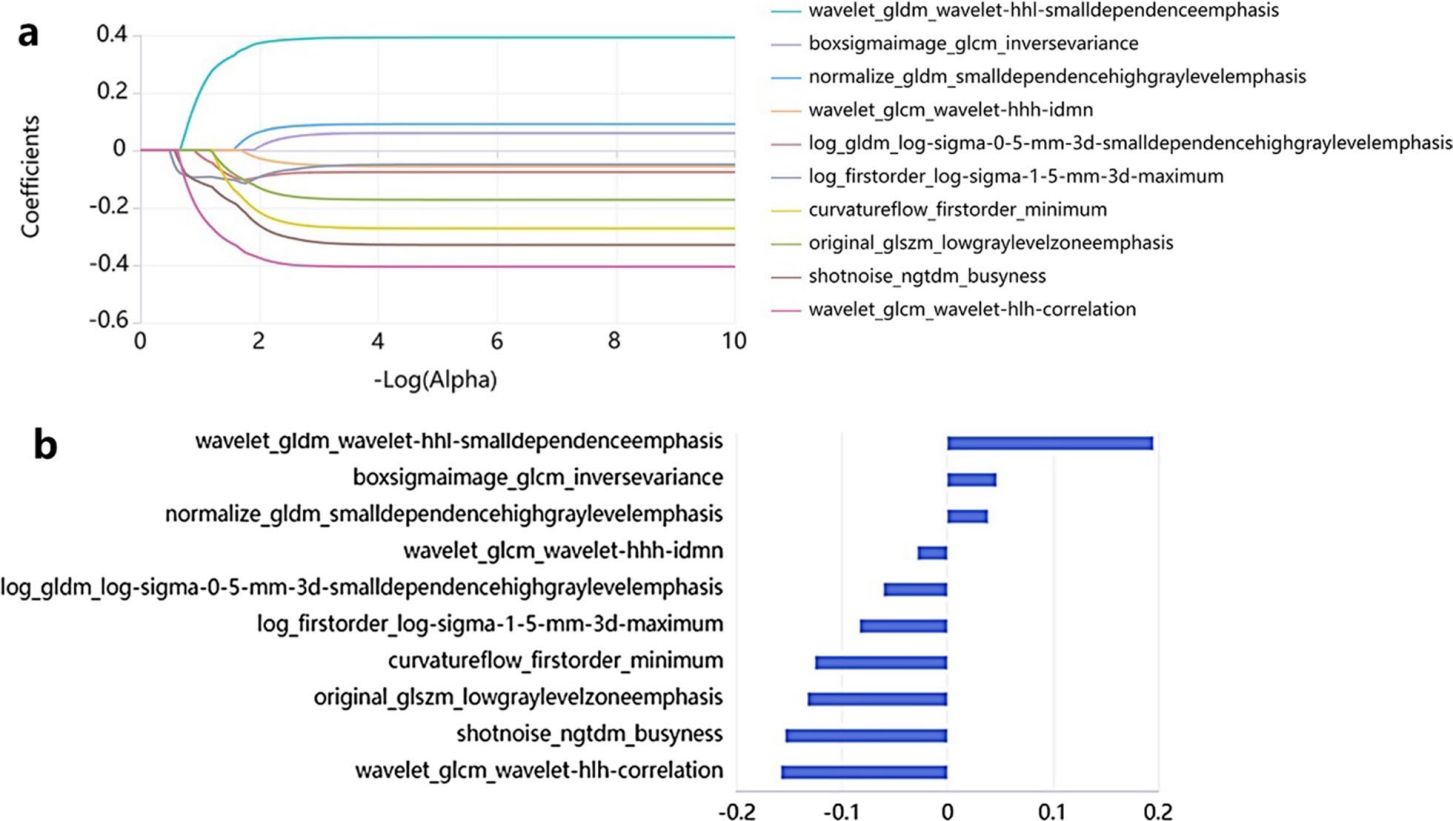
**a** LASSO coefficient of the features are drawn via fivefold cross-validation, and 10 radiomic features were remained, with an optimized hyperparameter α of 0.039. **b** Through LASSO logical regression analysis, 10 optimal radiation features are determined to calculate the RadScore value

Through LASSO regression analysis, the optimized hyperparameter α is 0.039, retaining 10 radiological features (Fig. 3). RadScore 's formula is as follows: RadScore = 0.195573032 * wavelet_gldm_wavelet-hhl-smalldependenceemphasis + 0.04672349 * boxsigmaimage_glcm_inversevariance + 0.03858112 * normalize_gldm_smalldependencehighgraylevelemphasis + -0.028275391 * wavelet_glcm_wavelet-hhh-idmn + -0.06043785 * log_gldm_log-sigma-0–5-mm-3d-smalldependencehighgraylevelemphasis + -0.0830874 * log_firstorder_log-sigma-1–5-mm-3d-maximum + -0.125622019 * curvatureflow_firstorder_minimum + -0.132682517 * original_glszm_lowgraylevelzoneemphasis + -0.153745949 * shotnoise_ngtdm_busyness + -0.157849476*wavelet_glcm_wavelet-hlh-correlation. The median RadScore was 0.75 ± 0.63, 1.03 ± 0.46, and 1.23 ± 0.29 in the unvaccinated, incompletely vaccinated, and completely vaccinated groups, respectively, and there was an overall statistically significant difference in RadScore across the three groups and pairwise comparisons. (Overall *P* < 0.001, unvaccinated group vs .partially vaccinated group *P* = 0.001, partially vaccinated group vs. fully vaccinated group *P* = 0.002, unvaccinated group vs fully vaccinated group *P* < 0.001).

### Fusion model

**Fig. 4:**
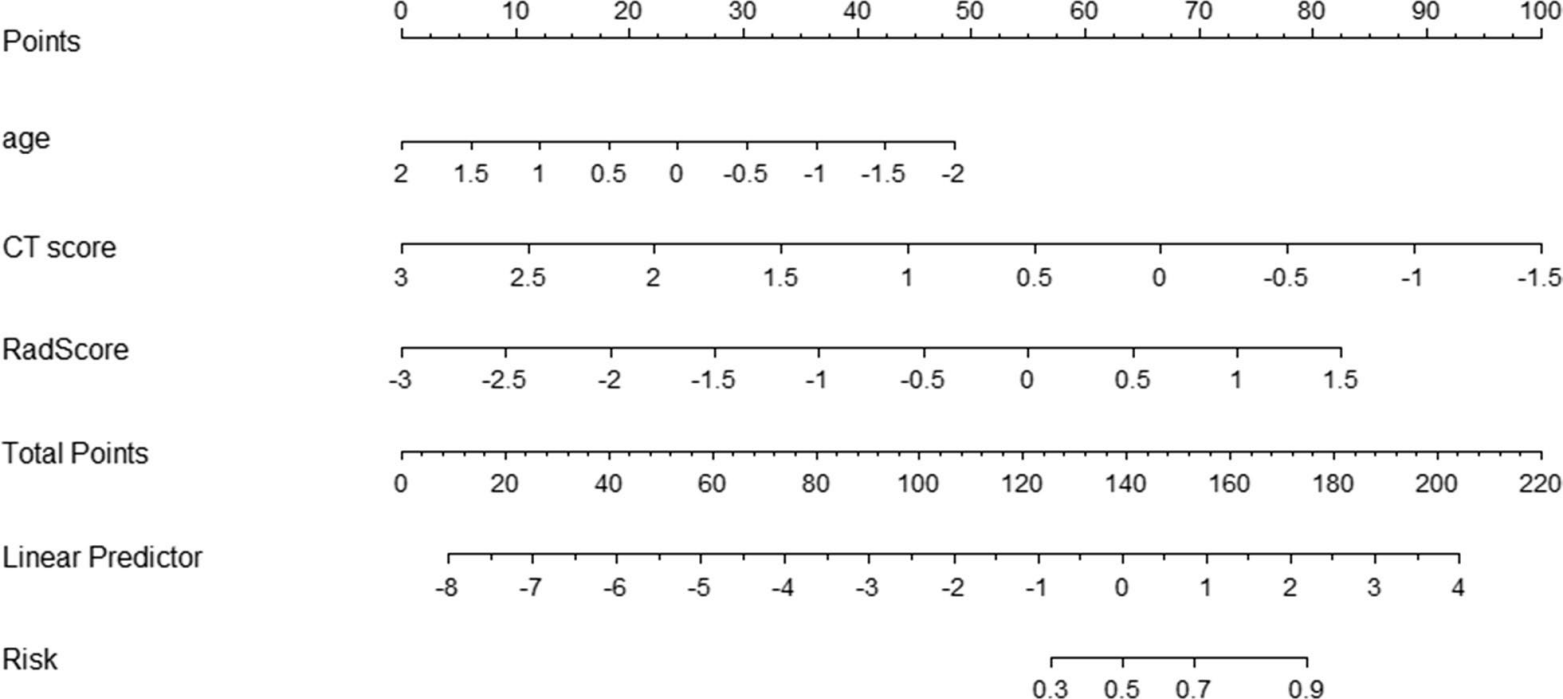
Nomogram for the multivariate logistic model between the partially vaccinated group and the unvaccinated group (reference level); (a): probability of partially vaccinated group rather than unvaccinated group

**Fig. 5:**
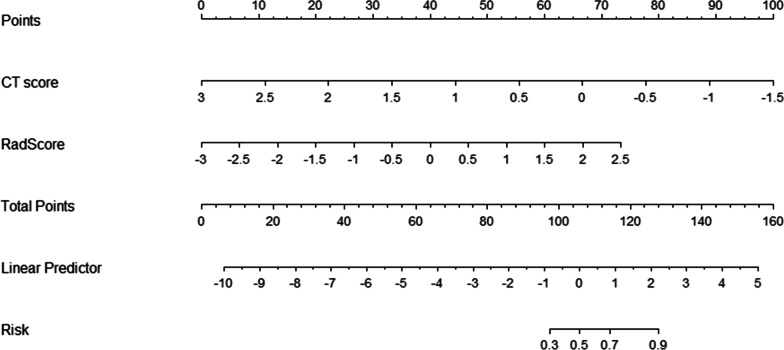
Nomogram for the multivariate logistic model between the full vaccinated group and the unvaccinated group (reference level); (b): probability of full vaccinated group rather than unvaccinated group

**Fig. 6:**
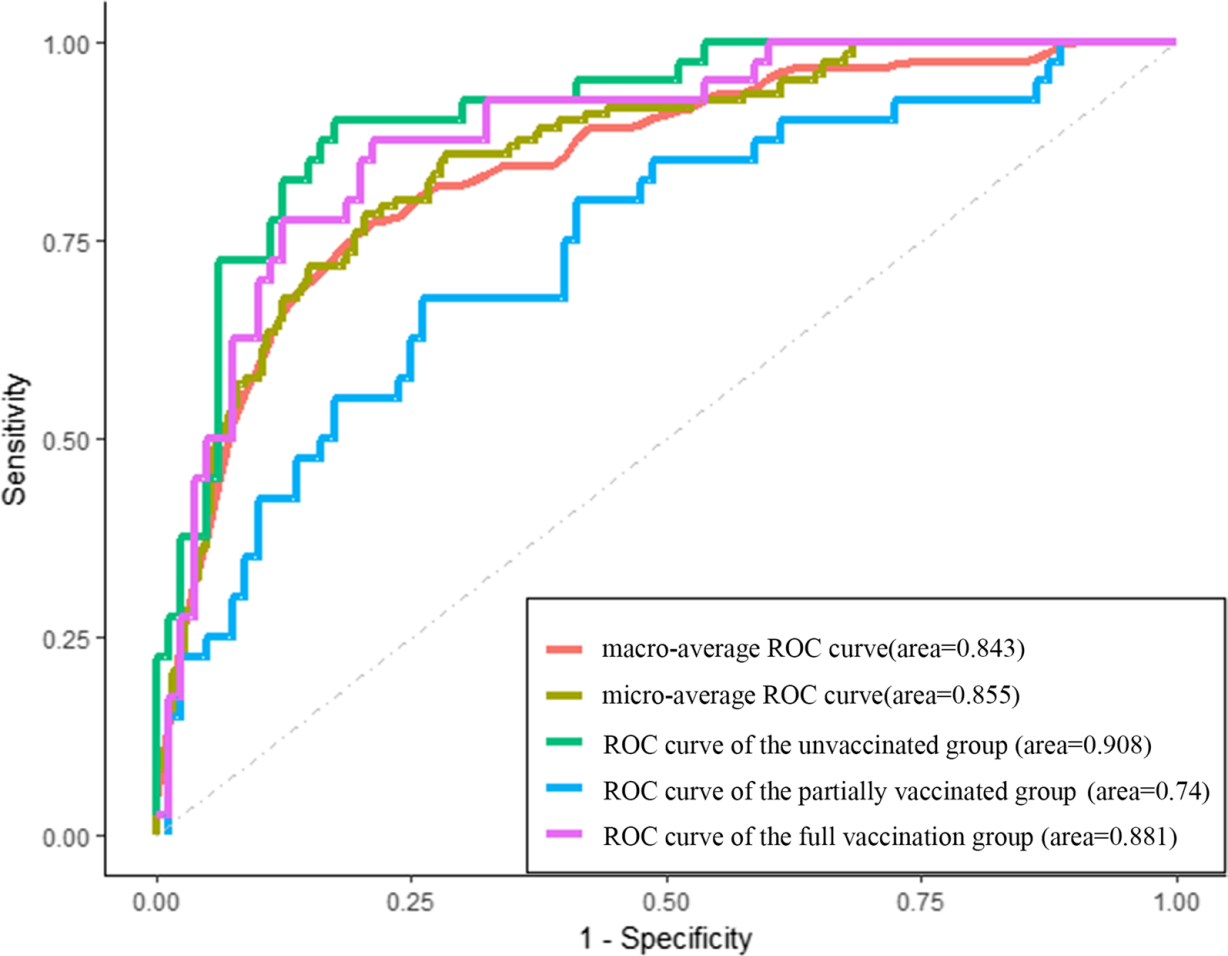
The fusion models predicted receiver operating characteristic (ROC) curves for the three groups by vaccination status. The ROC curves of the macro-average and micro-average AUC illustrate the overall discriminatory power of the model

After univariate logistic regression and multivariate logistic regression analysis, age, CT score and Radscore were included in the final combined model (Table 3, Fig. 4), and CT score and Radscore were included in the final combined model (Table 4, Fig. 5); All of the Hosmer–Lemeshow test for the combined model had a good fit (all *P* > 0.05). In addition, to further explain the combined model, we constructed Nomogram as detailed in Fig. 5. The fusion models yielded AUC values of 0.908, 0.74, and 0.881 for the unvaccinated group, the partially vaccinated group, and the fully vaccinated group, respectively. The overall prediction accuracy of the multivariate logistic model across the three groups was 0.70. The overall macro-average and micro-average AUC of the model were 0.843 and 0.855, respectively (Fig. 6).

**Table 3:** Predictive analysis of predictors in the multivariate logistic model between the partially vaccinated group and the unvaccinated group (reference level)

	Univariate analysis			Multivariate analysis		*p*
	OR	95% CI	*p*	OR	95% CI	
Age	**0.445**	**0.277**–**0.714**	**0.001**	**0.968**	**0.940**–**0.997**	**0.016**
White Blood Cell Count(× 109/L)	0.894	0.586–1.362	0.601			
Lymphocyte Count(× 109/L)	**1.962**	**1.110**–**3.468**	**0.020**			
CT score	**0.280**	**0.141**–**0.554**	** < 0.001**	**0.259**	**0.112**–**0.597**	**0.002**
RadScore	**2.296**	**1.346**–**3.917**	**0.002**	**2.940**	**1.471**–**5.879**	**0.002**

Statistically significant values are identified in boldface

**Table 4:** Predictive analysis of predictors in the multivariate logistic model between the full vaccinated group and the unvaccinated group (reference level)

	Univariate analysis			Multivariate analysis		
	OR	95% CI	*p*	OR	95% CI	*p*
Age	**0.595**	**0.384**–**0.920**	**0.020**			
White Blood Cell Count(× 109/L)	0.843	0.568–1.251	0.396			
Lymphocyte Count(× 109/L)	**2.255**	**1.295**–**3.927**	**0.004**			
CT score	**0.195**	**0.095**–**0.400**	** < 0.001**	**0.133**	**0.044**–**0.408**	** < 0.001**
RadScore	**2.730**	**1.605**–**4.643**	** < 0.001**	**3.933**	**1.699**–**9.100**	**0.001**

Statistically significant values are identified in boldface

## Discussion

Our study constructed nomograms based on radiomics and clinical factors for predicting vaccination status in patients with novel coronavirus disease. The results showed that CT score and RadScore were independent factors for predicting all vaccination status. Radiomics is mainly based on radiomics features of the degree of lung inflammation, and nomograms that combine clinical factors can well identify patient vaccination status. At present, some scholars have developed predictive models for evaluating patients with new coronary pneumonia, but the validation of the models needs to be further explored. Therefore, we adopted an internal validation approach of the model, which has been demonstrated [21].

In the unvaccinated group, compared with the partially vaccinated group and the full vaccination group, the age was significantly older and the peripheral blood lymphocyte count was lower. Studies have shown that the changes of lymphocytes are closely related to the pathogenesis of the virus [22]. SARS-CoV-2 infection can destroy lymph nodes by inhibiting bone marrow or directly inducing immunity, resulting in peripheral blood lymphocytopenia [23]. In our article, the lymphocyte count of the people who were not vaccinated with novel coronavirus vaccine decreased significantly, which suggested that compared with the people who had been vaccinated with novel coronavirus vaccine, the delta variant infection in this group may have more serious immune cell consumption and cellular immune function impairment. Moreover, it is reported that the decrease of peripheral blood lymphocyte count in the early stage of admission is one of the potential early clinical early warning indicators of COVID-19 's severe / critical tendency [24]. We also found that the lymphocyte count in the partial vaccination group was lower than that in the complete vaccination group (1.55 ± 0.70 vs. 1.87 ± 0.70, *p* < 0.05), which indicated that the lymphocyte count might be valuable in reflecting the severity of coronavirus pneumonia.

From the overall chest CT score analysis, the degree of lung infection was related to the degree of vaccination and the difference was statistically significant. This result further demonstrated the previous findings that booster injection on the basis of vaccination could increase the protective effect of COVID-19 vaccine in humans [25, 26]. Besides, we also conducted a preliminary study on the chest HRCT manifestations of patients infected with unvaccinated, partially vaccinated and completely inoculated delta mutants, revealing some major manifestations on chest CT images. First of all, whether vaccinated or not, exudative changes with or without local consolidation foci were common in positive chest CT, that is, multiple or single ground glass shadows could be accompanied by solid lesions, simple single shadows foci were rare, and thickened vascular bundles could be seen in some shadows. Secondly, the lesions in the lung can involve each lung lobe, but usually the lower lobes of both lungs are relatively common, and the lesions are mostly distributed in the periphery and / or subpleural of the lung, and simple central lesions are rarely seen, which may be related to the pathological mechanism of viral pneumonia invading the pulmonary parenchyma. For example, in the early stage, the lesions are easy to involve the terminal bronchioles and the parenchyma around the respiratory bronchioles, and then spread along the bronchovesicular bundle to the middle lung field [27]. Other signs included interstitial changes, HRCT showed fine reticular linear high-density shadows or stripe focus, and some of them showed "paving stone" changes. Finally, extrapulmonary manifestations of HRCT, such as pleural effusion or mediastinal enlarged lymph nodes, were rare in the three groups.

In this study, 3D-ROI image features extracted by radiomics technology can provide more image information than conventional 2D-ROI, because 3D-ROI can provide more complete features of infected lung volume, easier to capture heterogeneous information of lesions, and eliminate the influence of manual sketching, increasing the repeatability and reliability of radiomics features, so it has higher diagnostic efficiency [28].

After feature standardization, elimination of redundant features and LASSO feature selection, the radiological score RadScore was constructed, and the chest CT of patients infected with delta mutant was quantitatively analyzed to explore the relationship between RadScore and the vaccination status of patients. The final experimental results show that after feature selection, a total of 10 radiological features constitute RadScore, including 8 texture features and 2 first-order statistical features. Among the 10 radiological features that can predict the vaccination status of patients, 7 imaging features are negatively correlated with RadScore, including those obtained by wavelet filter, mean normalization filter, curvature filter, shot noise filter and Laplace filter post-processing, which can indicate the invasion degree of coronavirus to lung parenchyma. Among them, the larger the eigenvalue of the first-order statistical feature 3D maximum diameter (log_firstorder_log-sigma-1–5-mm-3d-maximum) processed by Laplace filter, indicates that the wider the area of infection, the greater the possibility of patients not being vaccinated, so it is negatively correlated with RadScore. In addition, three imaging features were positively correlated with RadScore, including the texture features processed by gray area size matrix and box filter, which represented the texture features of residual normal lung parenchyma, and their values could indirectly reflect the damage degree of lung parenchyma. We found that there were statistical differences in RadScore among the three groups of patients with different vaccination status. The RadScore of the unvaccinated group was the smallest, the RadScore of the incomplete vaccinated group was the second, and the RadScore of the complete vaccinated group was the largest. The results show that the imaging score RadScore has the ability to identify the vaccination of patients, and it also proves that the larger the RadScore, the greater the possibility of full vaccination, and the smaller the imaging characteristic value showing the size of the infected area in the corresponding RadScore. This result is also consistent with the related literature that COVID-19 vaccine has protective effect on the lungs [29–33].

In addition, our research has some limitations. First of all, retrospective studies may have some selection bias; secondly, the case comes from a single center, the sample size in each group is relatively small, and due to the limited clinical data, more blood biochemical indexes, such as viral load and neutralizing antibody titer, are not included, which is obviously an important issue to be explored in the future. Besides, we exclude patients with basic lung diseases and other basic diseases, and we need to further explore the impact of different basic diseases on COVID-19 vaccine in the future. Finally, the clinical symptoms of the patients included were not dynamically monitored and evaluated. Therefore, further prospective studies with large-scale, multicenter and longer observation periods are needed.

## Conclusion

To sum up, from clinical features, lung imaging features to radiomics analysis, we verified the protection of different COVID-19 vaccination states (unvaccinated, partially vaccinated, and fully vaccinated) on the lung, which is the easy attacking organ of delta mutant strain. At the same time, it affirmed the positive effect of COVID-19 vaccine on pulmonary inflammatory symptoms and lymphocyte count (immune system) during infection with delta variant strain, as well as the necessity of implementing COVID-19 vaccination and boost shots immunization strategy.

## Data Availability

The datasets used and/or analyzed during the current study available from the corresponding author on reasonable request.

## Abbreviations

COVID-19: Coronavirus disease
HRCT: High resolution computed tomography
3D-ROI: 3-Dimensional regions-of-interest
RadScore: The radiomics signature
RT-PCR: Still real-time reverse transcription polymerase chain reaction
WHO: World health organization
ANOVA: Analysis of variance
LASSO: Least absolute shrinkage and selection operator
ICC: Intra-group correlation coefficient
ROC: Receiver operating characteristic
AUC: Curve and the area under the curve
